# Prognostic value of lactate metabolism-related gene expression signature in adult primary gliomas and its impact on the tumor immune microenvironment

**DOI:** 10.3389/fonc.2022.1008219

**Published:** 2022-09-20

**Authors:** Zhihao Wang, Shuxin Zhang, Junhong Li, Yunbo Yuan, Siliang Chen, Mingrong Zuo, Wenhao Li, Wentao Feng, Mina Chen, Yanhui Liu

**Affiliations:** ^1^ Department of Neurosurgery, West China Hospital of Sichuan University, Chengdu, China; ^2^ Department of Head and Neck Surgery, Sichuan Cancer Hospital and Institute, Sichuan Cancer Hospital, School of Medicine, University of Electronic Science and Technology of China, Chengdu, China; ^3^ Neuroscience & Metabolism Research, State Key Laboratory of Biotherapy, West China Hospital of Sichuan University, Chengdu, China

**Keywords:** glioma, lactate metabolism, tumor microenvironment, immunity, prognosis

## Abstract

Glioma is one of the most malignant intracerebral tumors, whose treatment means was limited, and prognosis was unsatisfactory. Lactate metabolism patterns have been shown to be highly heterogenous among different tumors and produce diverse impact on the tumor microenvironment. To understand the characteristics and implications of lactate metabolism gene expression, we developed a lactate metabolism-related gene expression signature of gliomas based on RNA-sequencing data of a total of 965 patient samples from TCGA, CGGA, and our own glioma cohort. Sixty-three lactate metabolism-related genes (LMGs) were differentially expressed between glioma and normal brain tissue, and consensus clustering analysis identified two clusters distinct LMG expression patterns. The consensus clusters differed in prognosis, molecular characteristics and estimated immune microenvironment landscape involving immune checkpoint proteins, T cell dysfunction and exclusion, as well as tumor purity. Univariate Cox regression and Least Absolute Shrinkage and Selection Operator (LASSO) Cox hazard regression was applied in determining of prognosis-related lactate metabolism genes (PRLMGs), on which prognostic lactate metabolism risk score (PLMRS) was constructed. The high PLMRS group was associated with significantly poorer patient outcome. A nomogram containing PLMRS and other independent prognostic variables was established with remarkable predictive performance on patient survival. Exploration on the somatic mutations and copy number variations of the high- and low-PLMRS groups demonstrated their distinct genetic background. Together, our results indicated that the expression signature of LMG was associated with the prognosis of glioma patients and influenced the activity of immune cells in the tumor microenvironment, which may serve as a potential biomarker for predicting response of gliomas to immunotherapy.

## Introduction

Glioma accounts for over 80% of all primary malignant intracerebral tumors in adult. Glioblastoma (GBM) accounts for 58.4% of newly diagnosed adult primary gliomas and is the most aggressive glioma subtype (1). Despite comprehensive anti-cancer treatment involving surgical resection, followed by radiotherapy and temozolomide (TMZ) chemotherapy, the 5-year-survival of GBM remained 6.8%-9.8%, while the relative survival for patients with astrocytoma and oligodendroglioma were 43.5% and 77.9%, respectively (1, 2). Warburg effect describes a metabolic characteristics of cancer cells, whose energy is mostly provided through high level of glycolysis, regardless of richness of oxygen supply, which is also named as “aerobic glycolysis” (3). Lactate, produced by active glycolysis, has received much attention in cancer research in the past decades, and lactate metabolism has been identified as a hallmark of cancer, which contributes to tumorigenesis (4). Clinical investigations have connected high level of lactate in the tumor microenvironment (TME) with poor prognosis in cervical cancer, breast cancer and head and neck squamous cell carcinomas (5–7). A magnetic resonance spectroscopy (MRS) study found that lactate concentration was significantly higher in isocitrate dehydrogenase (IDH) wild-type gliomas than the less aggressive IDH mutants (8).

Recent studies have revealed the important role of tumor lactate metabolism in the tumor immune microenvironment (9). For example, lactate accumulation in TME results in downregulation of CD4+ T cells, natural killer (NK) and natural killer T cells and upregulation of T-regular cells (Tregs) and myeloid-derived suppressor cells (MDSCs), leading to suppression of immune response (10–13). On the other hand, elevated serum lactate dehydrogenase (LDH) predicts shorter survival in melanoma, non-small cell lung cancer (NSCLC) and esophageal squamous cell carcinoma patients treated with immune checkpoint inhibitors (ICIs) (14–16). Additionally, extracellular acidic microenvironment induced by lactate is more toxic for normal cells compared with tumor cells (17, 18). Duan et al. reported lactate metabolic characteristics in glioblastoma and found hypoxia inducible factor 1α (HIF-1α), monocarboxylate transporters 1 (MCT1) and 4 (MCT4) indicating poor prognosis (19).

Lactate metabolism-related gene (LMG) expression signatures have been used in prognosis prediction and microenvironment of breast cancer, lung adenocarcinoma and hepatocellular carcinoma (20–22). However, such analysis hasn’t been applied in glioma. In the present study, we aimed to understand the relationship between lactate metabolism and glioma TME with bioinformatics methods and attempted to investigate its clinical implication by constructing a prognostic LMG expression signature.

## Patients and methods

### Data acquisition and definition of lactate metabolism-related genes

RNA-sequencing and clinical data of adult primary gliomas and normal control samples were collected from The Cancer Genome Atlas (TCGA)^1^ database (TCGA-GBM and TCGA-LGG), the Chinese Glioma Genome Atlas (CGGA)^2^ together with glioma patients from West China Hospital (WCH). Sample collection and sequencing process are available in **Supplementary Materials Data Sheet 2**. R package “TCGAbiolinks” was used to download data from TCGA (23, 24). Patients under 18-years-old at the time of diagnosis were excluded. The Molecular Signature Database (MSigDB) was used to search for genes involved in lactate metabolism with the key word “lactate” (25). To make the data comparable, genes unavailable or expressed at very low level (maximum fragment per kilobase million, FPKM under 0.1) in either of the dataset were excluded. R package “limma” was used to identify differentially expressed genes (DEG) comparing gliomas and normal brain tissues in TCGA database. Genes with adjusted P-values <0.05 were considered significant DEGs. Lactate metabolism-related genes (LMGs) referred to the overlap between genes included in the lactate metabolism pathways gene sets curated in the MSigDB and the differentially expressed genes between gliomas and normal brain samples in the TCGA dataset.

### Unsupervised clustering analysis using LMGs

Consensus clustering analysis was applied to divide different lactate metabolism patterns based on expression of LMGs using R package “ConsensusClusterPlus” with 100 iterations (26). The optimal number of clusters was determined based on sample size of each cluster and the cumulative distribution function (CDF) curve of consensus index values in the consensus matrix (27). Briefly, we aimed to maximize sample size of each cluster while keeping a gradually increasing CDF. Principal component analysis (PCA) of all DEGs performed to visualize the difference in the transcriptome between clusters.

### Clinical characteristics and biological function of consensus clusters

DEGs between the two clusters identified in consensus clustering with |log2FC| >0.5 and adjusted P-values <0.05 were selected for functional analysis. In the gene sets curated in MSigDB, Kyoto Encyclopedia of Genes and Genomes (KEGG) and Hallmark gene sets (HALLMARK) were utilized to conduct Over-representation (ORA) and Gene set enrichment analysis (GSEA) using R package “clusterProfiler” (28). To analyze the differential expressed signaling pathways between the clusters, we first transformed the logFPKM matrix into pathway expression matrix using R package “GSVA” and identified differentially expressed pathways using “limma” (29).

### Tumor immune microenvironment landscape

Cibersortx analysis^3^ was used to estimate the fraction of infiltrating immune cells of each tumor based on the RNA-seq data (30). The Estimation of STromal and Immune cells in MAlignant Tumor tissues using Expression data (ESTIMATE) score was also utilized in analyzing the differences of tumor stromal and immune microenvironment (31).

For tumor purity estimates, we used the ESTIMATE tumor purity and consensus purity estimation (CPE) data published by Aran et al. (32). The Tumor Immune Dysfunction and Exclusion (TIDE) algorithm was used to evaluate T cell dysfunction score and T cell exclusion score (33).

### Identification of prognosis-related lactate metabolism genes

To further investigate the role of LMGs in glioma prognosis and construct a prognostic model, prognosis-related lactate metabolism genes (PRLMGs) were identified. Data in TCGA database were randomly split into training set and validation set at a ratio of 6:4. Genes with adjusted P-value <0.05 in univariate Cox regression were selected in following analysis. Least Absolute Shrinkage and Selection Operator (LASSO) Cox regression was performed in the training set to identify PRLMGs using R package “glmnet” (34). Genes whose coefficient was not 0 at lambda.min in 100 random repetition of LASSO Cox regression were defined as PRLMGs and selected for further analysis.

### Prognostic model and clinical analysis based on PRLMGs

To better understand the prognostic role of PRLMGs, a lactate metabolism related prognostic signature was established with PRLMGs. The formula of prognostic lactate metabolism risk score (PLMRS) was:


$$PLMRS\;score=\sum_{i=1}\left(Exp_{i}*coef_{i}\right)$$

Patients in training set were stratified into low-risk group and high-risk group accordingly. The optimal cut-off PLMRS was determined by function “surv_cutpoint” in R package “survminer” with a minimum group proportion of 0.3. Receiver operating characteristic (ROC) curves in validation sets of 1-year, 2-year and 3-year survival were illustrated and area under the ROC curve (AUC) were calculated by R package “timeROC”. Then, the prognostic model was tested in validation set.

### Establishment and validation of nomogram

R package “rms” was used to construct and evaluate the nomogram. The nomogram was constructed by training a Cox-regression model using the cph function in the rms package with PLMRS and other potentially prognostic clinical factors to predict patient survival at 1, 2, and 3 years after diagnosis. Calibration curve was calculated by bootstrapping using calibrate function in the rms package to compare the actual survival rate and nomogram-predicted survival probability at these time points. Corrected concordance index (C-index) was calculated using validate function from the “rms” package.

### Association between PLMRS and genetic alterations of gliomas

The somatic mutation and copy number variation (CNV) data of the glioma patients in the TCGA cohort was downloaded from cBioPortal^4^ (35). R package “maftools” were used to visualize most frequently mutated genes as well as PLMRGs (36). Gistic segment mean CNV values were used to generate the whole genome CNV profile. Amplification and homozygous deletion of genes were also reported in the cBioPortal data.

### Statistical analysis

R software^5^ (Version 3.6.1) was used for data analysis and visualization. For continuous variables student t test was performed in two groups to compare differences, and one-way ANOVA was conducted to compare between three or more groups. Chi-square test was performed to detect the differences of categorical variables. Survival analysis was performed by R package “survminer”. The comparison of each Kaplan–Meier (KM) curve was accomplished using the log-rank test. Coefficients and their significance in the univariate and multivariate Cox regression analyses were computed using the coxph function. As for the statistical results, a two-sided P value <0.05 was considered a significant statistical difference. In the figures, a * indicated P < 0.05, while ** P < 0.01, *** P < 0.001, **** P < 0.0001.

### Statement of ethics

Ethical review and approvement of this research were conducted by the Ethical Committee of Sichuan University. All principles in the Declaration of Helsinki (Ethic number: 2018.569) were strictly followed. Patients and their authorized trustees were informed and written informed consents were obtained before surgery.

## Results

### Identification of glioma LMGs

**Figure 1:**
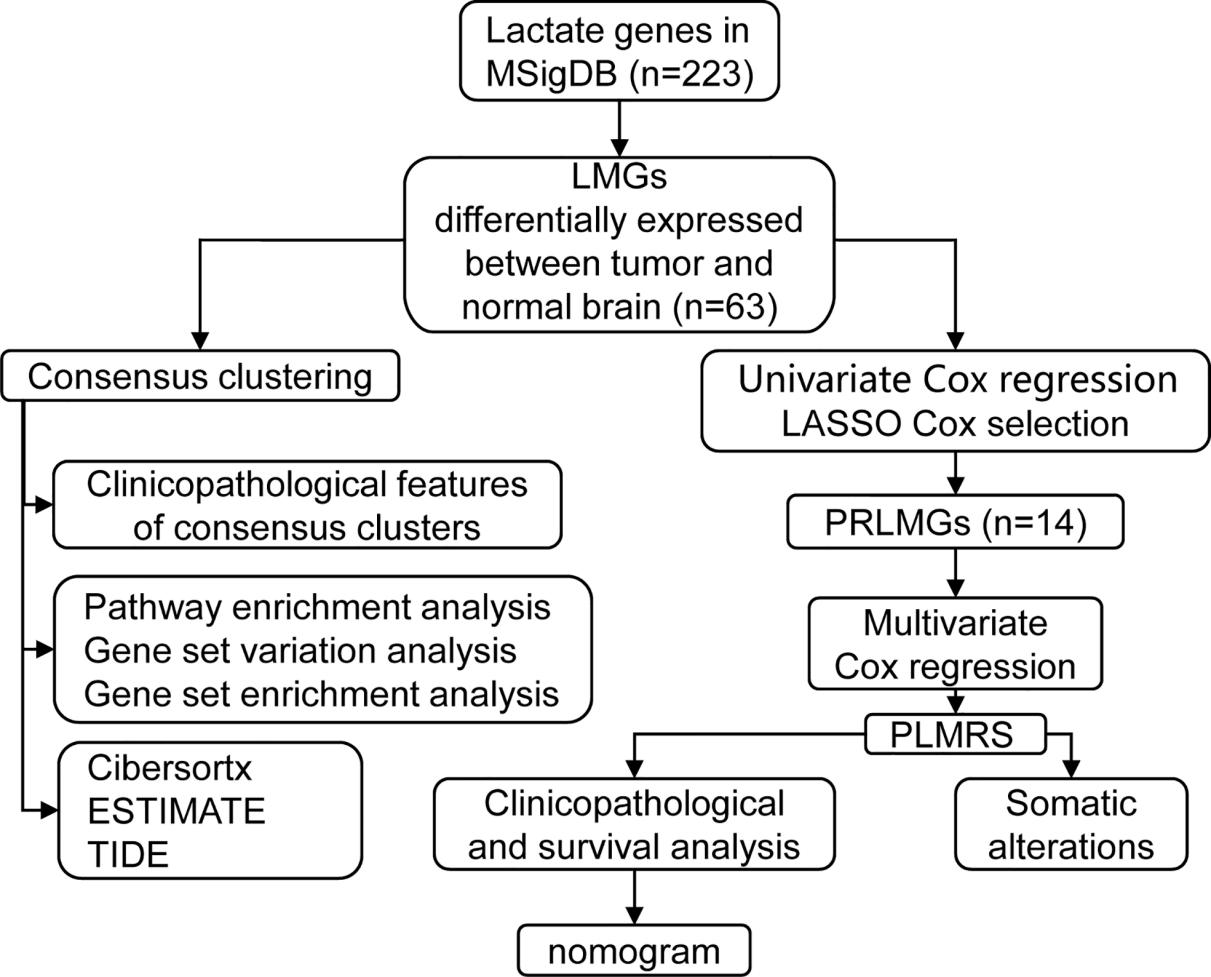
Flow chart of study design. MSigDB, Molecular Signature Database; DEG, differentially expressed gene; LMG, lactate metabolism-related gene; ESTIMATE, Estimation of STromal and Immune cells in MAlignant Tumor tissues using Expression data; TIDE, Tumor Immune Dysfunction and Exclusion; LASSO, Least Absolute Shrinkage and Selection Operator; PRLMG, prognosis-related lactate metabolism gene; PLMRS, prognostic lactate metabolism risk score.

Flow chart of the current study was shown in **Figure 1**. RNA-seq data of 662 adult primary gliomas and five normal brain samples were downloaded from TCGA, 226 gliomas from CGGA, and generated from 77 glioma samples and 16 normal brain samples from our own patient cohort. The clinical information of these patients was recorded in **Table 1**.

**Table 1:** Clinicopathological characteristics of adult primary glioma patients in TCGA, CCGA, and WCH cohort.

Sample size	TCGA	CGGA	WCH
Total Tumor	662	226	77
Normal Brain	5	–	16
Age	46 (18 - 89)	43 (18 - 79)	46 (19 - 77)
Gender			
Female	282	87	30
Male	380	139	47
Histology			
Astrocytoma	341	82	22
Oligodendroglioma	167	60	21
Glioblastoma	154	84	34
Grade			
G2	214	94	29
G3	237	48	14
G4	154	84	34
NA	57	0	0
IDH status			
Mutant	421	115	42
WT	236	110	35
NA	5	1	0
1p.19q.codeletion			
Codel	167	54	19
Non-codel	488	169	43
NA	7	3	15
TERT promoter status		–	
Mutant	340	–	30
WT	156	–	23
NA	166	–	24
MGMT promoter status			
Methylated	472	97	35
Unmethylated	157	115	13
NA	33	14	29
ATRX status		–	
Mutant	192	–	22
WT	459	–	53
NA	11	–	2

TCGA, The Cancer Genome Atlas; CGGA, Chinese Glioma Genome Atlas; WCH, West China Hospital; IDH, isocitrate dehydrogenase; TERT, telomerase reverse transcriptase; MGMT, O6-methylguanine-DNA methyltransferase; ATRX, Alpha Thalassemia Developmental Delay; WT, wild type; NA, not available.

**Figure 2:**
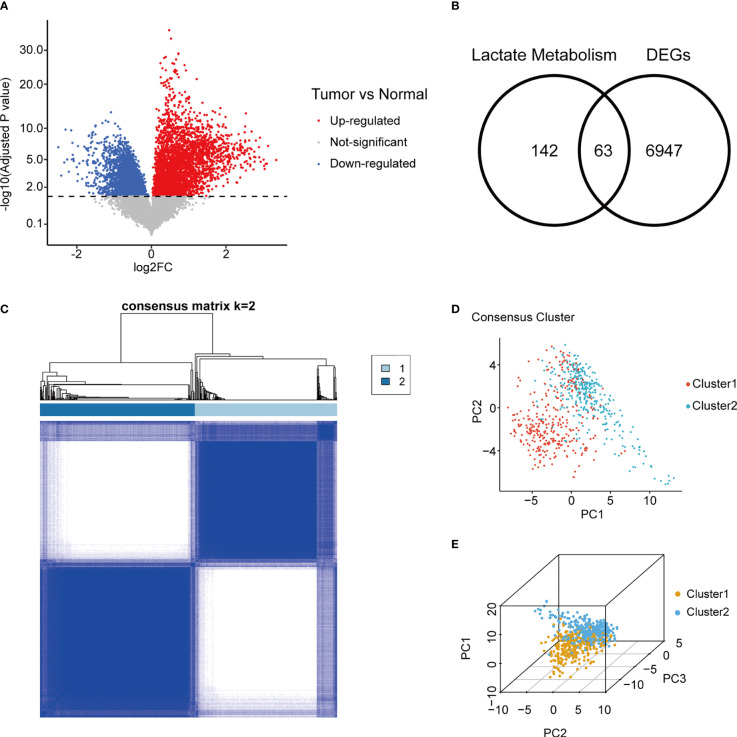
Consensus clustering of gliomas using LMG expression. **(A)** Volcano plot of gene fold change between adult primary glioma samples and normal brain in TCGA. **(B)** Overlap between tumor/normal DEGs and lactate metabolism related genes. **(C)** Consensus matrix of two LMGs clusters. **(D, E)** Concordance between two and three dimensional PCA of LMG expression and consensus clusters. LMG, lactate metabolism-related gene; TCGA, The Cancer Genome Atlas; DEG, differentially expressed gene; PCA, principal component analysis.

A total of 223 genes related to lactate metabolism were found in MSigDB, of which 205 genes were included (**Table S1**). In the TCGA cohort, 7010 genes were differentially expressed in mRNA level between glioma samples and normal brain sample (**Figure 2A**; **Table S2**). The 63 genes overlapped between DEGs, and lactate metabolism gene sets were defined as LMGs (**Figure 2B**).

### Consensus clustering of gliomas using LMG expression

Unsupervised consensus clustering was performed using RNA expression of LMGs in TGCA gliomas. Evaluation of the cluster sizes and CDFs found that the gliomas could be classified into two consensus clusters (**Figure 2C**; **Figure S1**). The two distinct LMG expression patterns of gliomas were also verified using two and three dimensional PCA (**Figures 2D, E**).

**Figure 3:**
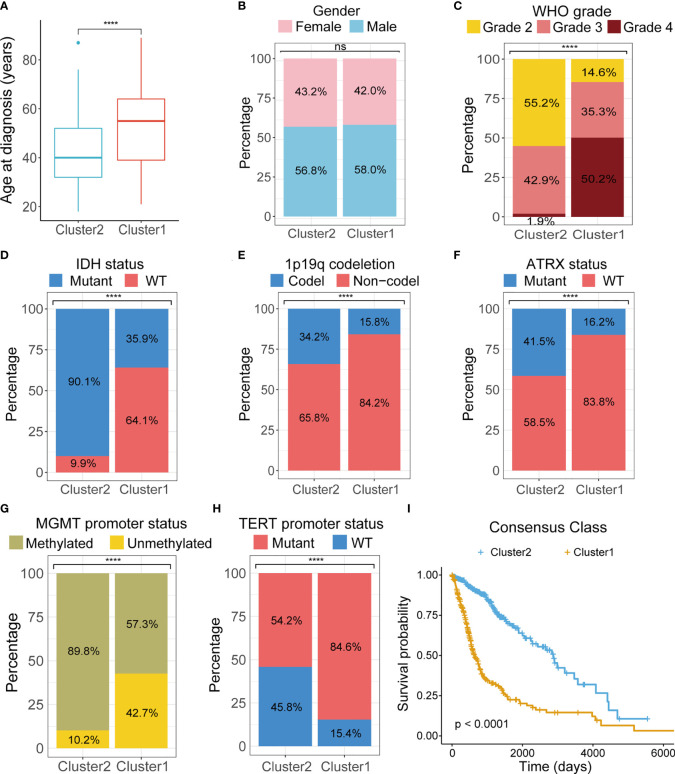
Comparison of clinicopathological characteristics between two clusters. Differences between two clusters were compared in age at diagnosis **(A)**, gender **(B)**, tumor grade **(C)**, IDH mutations **(D)**, 1p19q codeletion **(E)**, ATRX mutation **(F)**, MGMT promoter methylation **(G)**, TERT promoter mutation **(H)**. **(I)** K-M survival curves of two clusters. TCGA, The Cancer Genome Atlas; WHO, World Health Organization; IDH, isocitrate dehydrogenase; ATRX, Alpha Thalassemia Developmental Delay; MGMT, O6-methylguanine-DNA methyltransferase; TERT, telomerase reverse transcriptase; K-M, Kaplan–Meier; WT, wild type. ****p < 0.0001. ns, not significant.

To investigate the stratification, we combined clinicopathologic and survival information with consensus clusters. We found that clustering of gliomas was significantly related to patients’ age at diagnosis, WHO grade, IDH mutational status, 1p19 codeletion status, Alpha Thalassemia Developmental Delay (ATRX) mutational status, O6-methylguanine-DNA methyltransferase (MGMT) promoter methylation status, and telomerase reverse transcriptase (TERT) promoter mutational status, but not gender (**Figures 3A–H**). K-M curve also revealed significant difference in patient outcome between the two clusters (**Figure 3I**).

**Figure 4:**
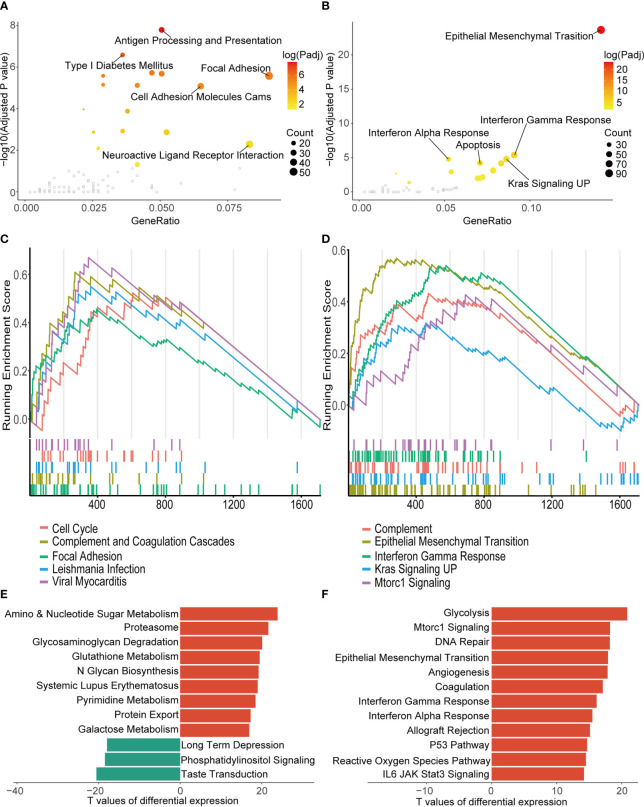
Biological function analysis of DEGs between the two consensus clusters. **(A, B)** Top five over-represented pathways in KEGG **(A)** and HALLMARK **(B)** annotation of the DEGs. **(C, D)** Top five pathways with the highest normalized enrichment score of GSEA in the KEGG **(C)** and the HALLMARK gene sets **(D)**. **(E, F)** Ten pathways with smallest adjusted p-value of GSVA in KEGG **(E)** and HALLMARK pathways **(F)**. TCGA, The Cancer Genome Atlas; KEGG, Kyoto Encyclopedia of Genes and Genomes; GSEA, gene set enrichment analysis; UP, upregulation; GSVA, gene set variation analysis.

Functional analyses of DEGs between the two clusters found cell adhesion, immune response and epithelial mesenchymal transition (EMT) were over-represented in the KEGG and HALLMARK pathway annotation of these genes (**Figures 4A, B**). At the same time, in the GSEA, significant enrichment of the DEGs in cell cycle (normalized enrichment score = 2.564, adjusted-P value = 0.007) and EMT (normalized enrichment score = 4.413, adjusted-P value = 0.004) was also observed in the top 5 enriched gene sets of the KEGG and HALLMARK databases (**Figures 4C, D**). GSVA of two clusters showed enhanced expression of genes involved in the typical anabolic pathways of malignant tumors in the more aggressive cluster 1 (**Figures 4E, F**).

### Differential TME immune characteristics between consensus clusters

**Figure 5:**
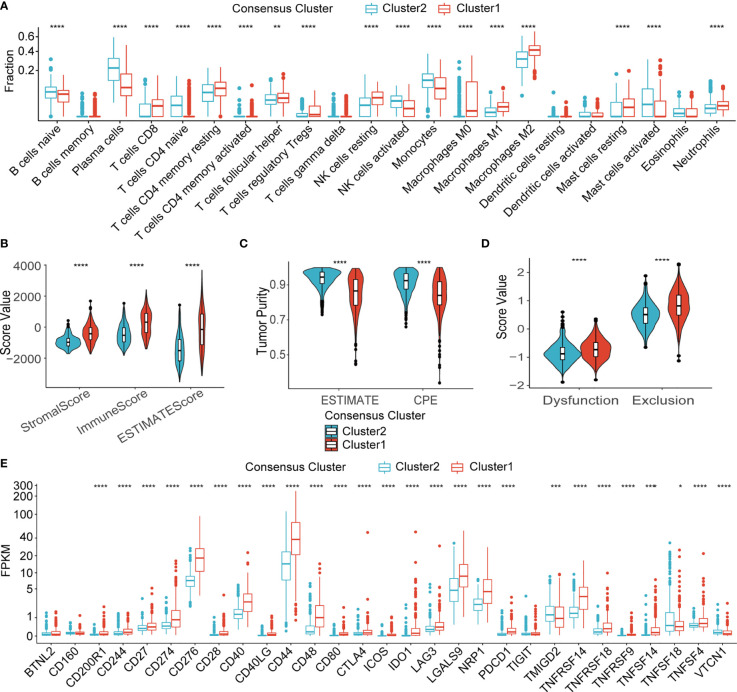
Immune microenvironment of two clusters. **(A)** Fraction of 22 types of immune infiltrating cells in Cibersortx in two clusters. **(B)** ESTIMATE score of two clusters. **(C)** Tumor purity estimation of two clusters using the ESTIMATE and the CPE algorithms **(D)** T cell dysfunction score and T cell exclusion score of two clusters. **(E)** mRNA expression of 29 ICPs in two clusters. TCGA, The Cancer Genome Atlas; NK, natural killer; ESTIMATE, Estimation of STromal and Immune cells in MAlignant Tumor tissues using Expression data; CPE, consensus measurement of purity estimations. *P < 0.05; **P < 0.01; ***P < 0.001; ****P < 0.0001.

*In silico* analysis of immune cell infiltration using Cibersortx revealed a number of significant differences between the LMG expression clusters (**Figure 5A**). More specifically, in the more aggressive cluster 1 gliomas, the proportion of CD8+ T cells, which were the main target of ICIs, were higher, while that of Tregs, which have immunosuppressive properties, was also higher. In the meantime, higher fraction of resting NK cells was predicted to reside tumors in cluster 1. Additionally, higher stromal cell and immune cell contents were also found in the cluster 1 tumor by the ESTIMATE algorithm (**Figure 5B**). On the other hand, tumor purity, which was negatively correlated with the ESTIMATE scores was lower in cluster 1 (**Figure 5C**). In concordance with the TME immune cell content estimations, prediction of T cell exclusion and functional status using the TIDE algorithm revealed a higher T cell dysfunction and exclusion score in cluster 1, which suggests a potentially more complicated immune background in the cluster 1 gliomas and that these tumors were less likely to respond to immunotherapy in contrast to cluster 2 (**Figure 5D**). To shed light on the possible immune pathways that may interact with lactate metabolism, expression of 29 immune checkpoints (ICPs) mRNA was compared in TCGA database (**Figure 5E**) (37). The results indicated that most representative ICPs were significant higher in cluster 1, which further implied an immunosuppressive environment in cluster 1.

### Prognostic value of LMG expression signature

**Figure 6:**
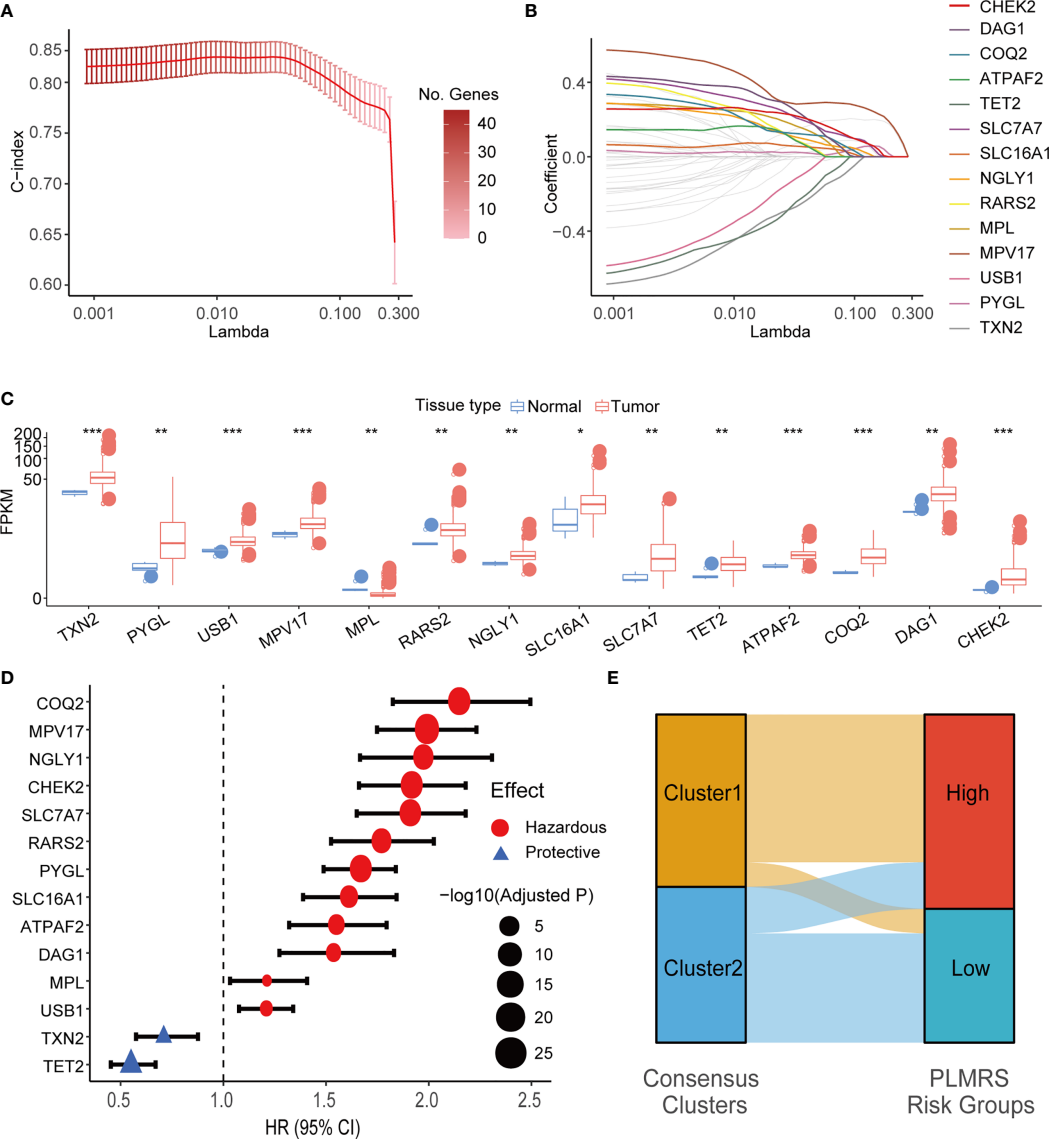
LASSO Cox regression and identification of PRLMGs. **(A)** Average of means and 95% confidence intervals of the C-index of LASSO Cox regression model at each lambda value. **(B)** Average of coefficients of LMGs in the LASSO Cox regression model at each lambda value. **(C)** mRNA expression of each PRLMG in glioma and normal brain tissue. **(D)** Univariate Cox regression analysis of PRLMGs in glioma prognosis. **(E)** Alluvial plot showing relationships between LMGs clusters and PLMRS risk groups. LASSO, Least Absolute Shrinkage and Selection Operator; C-index, concordance index; LMG, lactate metabolism-related gene; PRLMG, prognosis-related lactate metabolism gene. *P < 0.05; **P < 0.01; ***P < 0.001.

To understand if the LMG expression profile of gliomas could be utilized to evaluate prognosis of glioma patients, we attempted to construct an LMG-based prognostic measurement. We first split the TCGA glioma samples into training and testing datasets at a ratio of 6:4. In the training set, 63 LMGs were screened for their significant prognostic value using univariate Cox regression analysis. Then, 47 LMGs with adjusted P-value < 0.05 in univariate Cox regression were used to build multivariate LASSO Cox regression models for 100 random repetitions to select LMGs that showed unconfounded robust correlation with patient survival. In the end, 14 genes had non-zero coefficients in 100 LASSO Cox regression models and were qualified as prognostic LMGs (PRLMGs, **Figures 6A, B**).

Expression of PRLMGs was listed in **Figure 6C**. Among these genes, 13 of them had higher expression in tumor tissues, while MPL was expressed at a lower level in gliomas than the normal brain tissues (**Figure 6C**). In univariate cox regression analysis, 12 of PRLMGs were associated with worse prognosis, while the rest two were associated with prolonged OS (**Figure 6D**). All the PRLMGs were used to construct a final Prognostic LMG expression Risk Score (PLMRS) using multivariate Cox regression in the TCGA training set. The final calculation formula was:


$$\begin{array}{c} \text{PLMRS}=1.806^{*}\text{MPL}+0.306^{*}\text{ATPAF}2+0.297^{*}\text{CHEK}2+0.204^{*}\text{NGLY}1+0.104^{*}\text{COQ}2+0.088^{*}\text{SLC}7\text{A}7+0.071^{*}\text{MPV}17+0.048^{*}\text{RARS}2+0.034^{*}\text{DAG}1 \end{array}\begin{array}{c} +0.011^{*}\text{PYGL}+0.008^{*}\text{SLC}16A1-0.027^{*}\text{TXN}2-0.181^{*}\text{USB}1-0.517^{*}\text{TET}2. \end{array}$$

**Figure 7:**
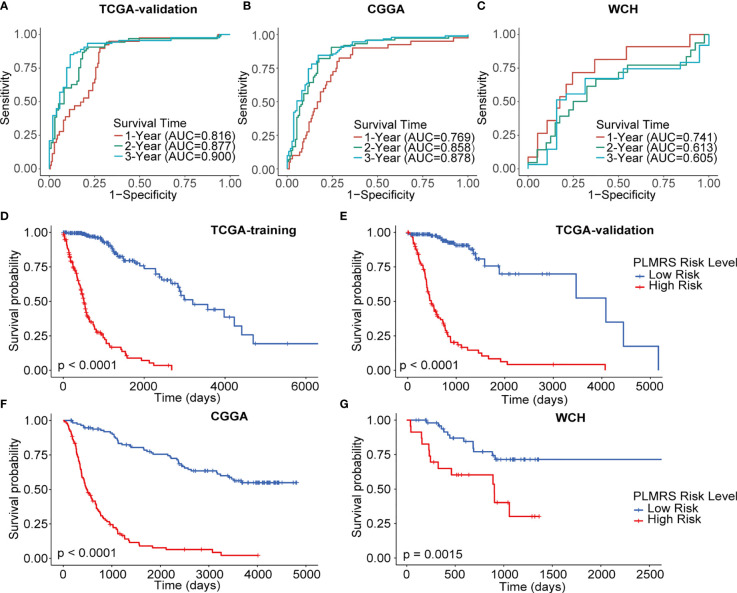
Prognostic value of PLMRS. ROC curves and matched AUC of 1-, 2-, 3-year survival in TCGA validation group **(A)**, CGGA **(B)** and WCH **(C)**. K-M curves of high-risk and low-risk groups in TCGA training group **(D)**, TCGA validation group **(E)**, CGGA **(F)** and WCH **(G)**. TCGA, The Cancer Genome Atlas; CGGA, Chinese Glioma Genome Atlas; WCH, West China Hospital; PLMRS, prognostic lactate metabolism risk score; ROC, receiver operating characteristic curve; AUC, area under the curve.

Using optimal PLMRS cutoff determined by maximizing outcome difference in each cohort (1.76 for the TCGA cohort, 8.79 for the CGGA cohort, 0.042 for our own cohort), the gliomas was divided into low- risk and high-risk groups. An alluvial diagram demonstrated the gliomas in cluster 1 were more likely to be at the high-risk group (**Figure 6E**). ROC analysis found that the PLMRS showed high predictive performance for 1-, 2-, 3-year survival in not only the TCGA-validation group, but also the CGGA and our own patient cohort (**Figures 7A–C**). In each cohort, patients in the low-risk group had significantly better outcome compared to those in the high-risk group (**Figures 7D–G**).

### Development and validation of a PLMRS-based nomogram

**Figure 8:**
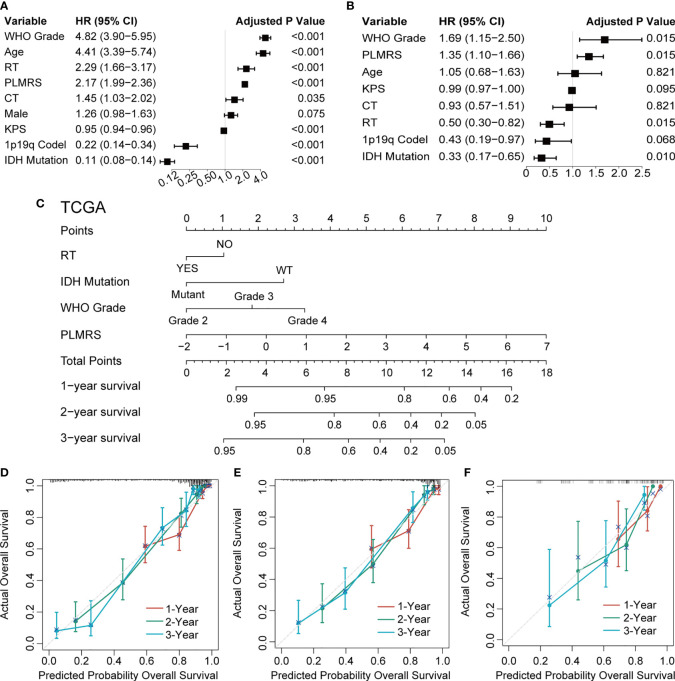
Nomogram construction and validation. Univariate **(A)** and multivariate **(B)** Cox regression to identify independent prognostic factors. **(C)** Nomogram of 1-, 2-, 3-year survival of glioma established in TCGA. Calibration plots of nomogram developed by TCGA **(D)**, CGGA **(E)** and WCH **(F)**. TCGA, The Cancer Genome Atlas; CGGA, Chinese Glioma Genome Atlas; WCH, West China Hospital; WHO, World Health Organization; PLMRS, prognostic lactate metabolism risk score; RT, radiotherapy; CT, chemotherapy; KPS, Karnofsky Performance Score; IDH, isocitrate dehydrogenase; WT, wild type.

To demonstrate the translational value of PLMRS in clinical settings, we attempted to construct a nomogram that involved the PLMRS and potential prognostic clinical information. Univariate Cox regression analysis of the TCGA cohort found that apart from the PLMRS, the age and Karnofsky Performance Score (KPS) of patients at diagnosis, the WHO grade, codeletion of chromosome arms 1p and 19q, mutational status of IDH genes, as well as chemotherapy and radiotherapy were significantly associated with patient survival (**Figure 8A**). In further multivariate analysis (**Figure 8B**) tumor grade, PLMRS, IDH mutational status and radiotherapy remained significant prognostic factors. As a result, we included these factors in the final nomogram construction (**Figure 8C**; **Figure S2**). The calibration plot of the nomogram for each cohort showed that the prediction of the nomogram was highly consistent with observed patient outcome in all three cohorts (**Figures 8D–F**). Compared with PLMRS alone, nomogram combining PLMRS, and other independent prognostic predictors showed superiority in their predictive performance of patient prognosis (C-index 0.837 for PLMRS only vs. 0.851 for combined nomogram, TCGA; 0.771 vs. 0.787, CGGA; 0.627 vs. 0.699, WCH).

### Associations between PLMRS and characteristics of gliomas

**Figure 9:**
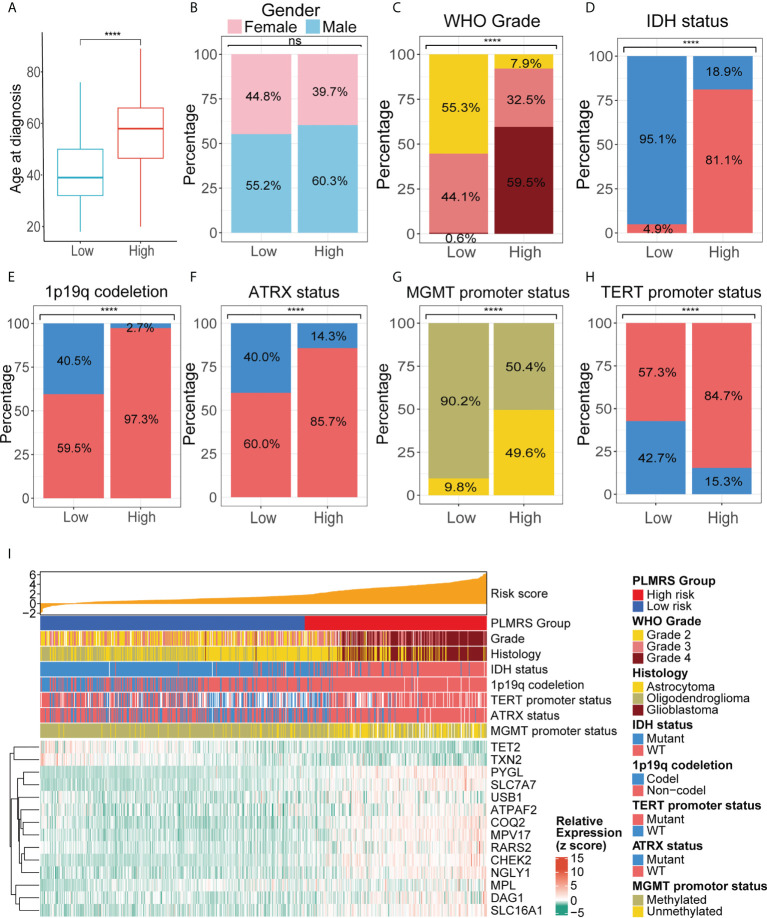
Comparison of clinicopathological characteristics in two PLMRS risk groups. Differences between the high and low risk groups were compared in age at diagnosis **(A)**, gender **(B)**, tumor grade **(C)**, IDH mutations **(D)**, 1p19q codeletion **(E)**, ATRX mutation **(F)**, MGMT promoter methylation **(G)**, TERT promoter mutation **(H)**. **(I)** Heatmap summarizing the clinicopathological feature and expressing PRLMGs in two risk groups. PLMRS, prognostic lactate metabolism risk score; TCGA, The Cancer Genome Atlas; WHO, World Health Organization; IDH, isocitrate dehydrogenase; ATRX, Alpha Thalassemia Developmental Delay; MGMT, O6-methylguanine-DNA methyltransferase; TERT, telomerase reverse transcriptase; WT, wild type. ****p < 0.0001. ns, not significant.

To understand how gliomas from two risk groups differed from each other, we analyzed the relationships between PLMRS and the clinicopathological features, as well as genetic background of gliomas. PLMRS was significantly associated with age at diagnosis, tumor grade, IDH mutational status, 1p19q codeletion status, ATRX mutational status, MGMT promoter methylation status, TERT promoter status and histology, but not gender (**Figures 9A–H**). Inspection at the expression of PRLMGs and the clinicopathological characteristics revealed apparent difference in these genes between different glioma subtypes (**Figure 9I**).

**Figure 10:**
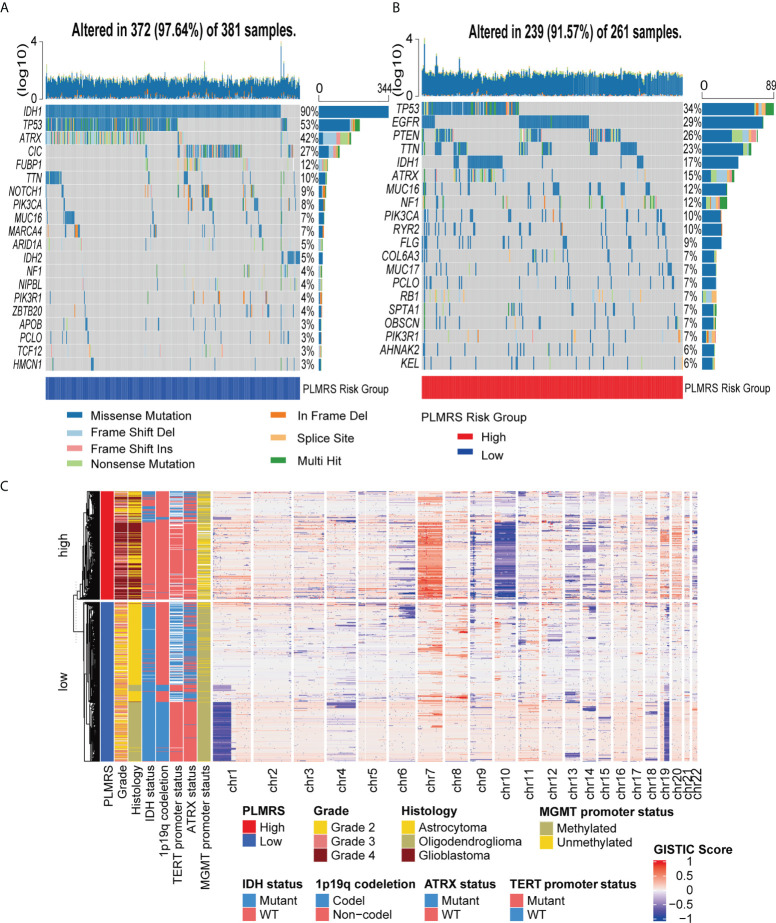
Somatic mutations and CNV of two risk groups. Top 20 somatic mutations in low-risk group **(A)** and high-risk group **(B)**. **(C)** Landscape of autosomal CNV in two risk groups. CNV, copy number variation; TCGA, The Cancer Genome Atlas; PLMRS, prognostic lactate metabolism risk score; IDH, isocitrate dehydrogenase; TERT, telomerase reverse transcriptase; ATRX, Alpha Thalassemia Developmental Delay; MGMT, O6-methylguanine-DNA methyltransferase; GISTIC, Genomic Identification of Significant Targets in Cancer.

Next, we analyzed the differences in the genetic alterations between the two risk groups by comparing the top 20 somatic mutations of low-risk group and high-risk group (**Figures 10A, B**). While a high rate of TP53 mutations were present in both groups, IDH1 mutation occurred in 90% of gliomas in low-risk group, but less than 20% in high-risk group. EGFR was the second most common mutations in high-risk group. At larger genomic landscape, (**Figure 10C**), high-risk gliomas exhibited evident gain of chromosome 7 and coexisting loss of chromosome 10.

## Discussion

Glioma is the most common primary malignant intracerebral tumor in adult (1). Despite the massive research investment and clinical experiments reported worldwide, the treatment of glioma has not yet to be revolutionized and the prognosis of glioma patients is not ideal, causing loss of quality of life (QOL) and financial stress for patients and their family (38, 39). Lactate is one of the components in the TME, whose concentration has been associated with the molecular features and prognosis of gliomas (8). Meanwhile, lactate metabolism has been considered to regulate tumor immunity through diverse mechanisms. With the assistance of public resources and our in-house dataset, we interrogated the relationships between the expression of lactate metabolism-related genes and clinicopathological characteristics of gliomas, as well as the potential impact of LMG expression on the landscape of glioma immune TME.

For tumor cells, aerobic glycolysis was considered more of a survival strategy than a compromise to insufficient oxygen supply. Nicotinamide adenine dinucleotide (NADH), the intermediate product of glycolysis, was used in synthesis of other organic molecules, while lactate was released, thus creating an acidic microenvironment (40). In gliomas, elevated lactate was associated with higher blood volume, and lower pH was connected with higher mitotic index (41, 42). Some researchers proposed a dynamic equilibrium of lactate in TME, where a group of tumor cells utilize lactate as energy source (43). In this study, we discovered that the unsupervised clustering with LMG expression signature could stratify glioma samples into two consensus clusters, in which one was associated with IDH wildtype gliomas and was more likely to have a more sophisticated and immunosuppressive microenvironment. This result was consistent with the findings reported by Wenger et al. that the higher level of glycolysis in the IDH wild type gliomas was associated with more lactate in TME, which could potentially disturb the distribution and functionality of immune cells (8).

Tumor immunotherapy has gained growing attention in the recent years. Current immunotherapies include cytokine therapy, ICIs, chimeric antigen receptor (CAR) T-cell therapy, oncolytic virus therapy, vaccine therapy and therapies targeting other immune related molecules or cells. However, none of the treatment showed promising clinical effect on gliomas so far (44). Among the numerous factors interfering anti-glioma immunity, lactate in the TME could play an important role in suppressing anti-tumor immune, by inducing Tregs and TAM M2, inhibiting NK and dendritic cells, activating MDSCs and decreasing cytotoxic T lymphocytes (CTLs) (45). Here, we investigated the association between lactate metabolism and the disturbed immune microenvironment of gliomas. In our result, both indolamine 2,3-dioxygenase 1(IDO1) and programmed cell death 1 ligand 1 (PD-L1 or CD 274) mRNA expression were higher in cluster 1. Overexpression of IDO1 resulted in recruitment of Tregs (46). This finding was consistent with the higher level of Tregs in cluster 1. PD-L1 expression was significant higher in TAM of GBM (47). Shan et al. reported that the expression of PD-L1 protein in TAM M2, which tended to be pro-tumor, was upregulated when treated with lactate (48). As expected, a higher fraction of TAM M2 score was observed in cluster 1. Programmed Cell Death 1 (PDCD-1 or PD-1) and Cytotoxic T-Lymphocyte Associated Protein 4 (CTLA4) were well-characterized ICPs. Inhibition of PD-1 leading to rescue of T lymphocyte and NK cell function, and CTLA-4 block resulted in activation of T cells (44). In our findings, both PD-1 and CTLA4 mRNA expression were elevated in cluster 1. In the meantime, NK cells were less activated in cluster 1. Although CD8+ T cells were higher in cluster 1, they could be inactivated due to the co-stimulation of PD-1 and CTLA4. In summary, our results suggest that the anti-tumor immunity is closely connected with the expression profile of LMGs.

To understand the clinical implications of LMG expression, we identified the LMGs exhibiting robust correlation with the clinical outcome of glioma patients, which we defined as PRLMGs. Among the PRLMGs, solute carrier family 7 member 7 (SLC7A7) and glycogen phosphorylase, liver form (PYGL) has been previously reported to be predictor of poor survival of GBM (49, 50). Solute carrier family 16 member 1 (SLC16A1), also known as monocarboxylate transporter 1 (MCT1), is responsible for the transmembrane delivery of lactate. Overexpression of SLC16A1 also predicted poor survival of high-grade gliomas (51, 52). The therapeutic and prognostic role of SLC16A1 has also been pointed out (53, 54). Tet methylcytosine dioxygenase 2 (TET2) catalyzed conversion of 5-methylcytosine (5mC) to 5-hydroxymethylcytocine (5hmC), which is important in homeostasis of hematopoietic cells (55). Of note, IDH mutation reversibly sabotaged the demethylation function of TET2 *via* oncometabolite 2-hydroxyglutarate (2HG) in acute myeloid leukemia (AML) (56). Similar mechanisms were reported in cholangiocarcinoma, mutation of IDH caused repression of TET2, which could induce immune suppression (57). In GBM, loss of TET2 was associated with poor survival, which could be mediated by sex determining region Y-box transcription factor 2 (Sox2) (58). Together, our results and previous findings indicate that, among the PRLMGs, SLC16A1 and TET2 are potentially important targets for intervention of glioma lactate metabolism that deserve priority in future investigation.

Despite the correlations between the expression of PRLMGs and aggressiveness of gliomas, a number of prognostic factors were also known to produce significant impact on the outcome of glioma patients. Besides patient comorbidity status and histological tumor grading, mutation of IDH genes and codeletion of chromosome arm 1p/19q have been well-recognized indicators for patient prognosis (59). Compared to the genetic background of gliomas, adjuvant treatments appeared to produce less impact on patient survival, but our results indicate that radiotherapy remained significant in prolonging patient survival. To translate this panel of clinical, pathological, and molecular information into succinct measurement, we combined the PLMRS with other independent prognostic factors to construct a nomogram that could accurately predict patient survival. Of note, in both the TCGA and CGGA cohort, PLMRS contributed most to the output of the nomogram, which emphasized the potential clinical value of the LMG expression signature in predicting outcome of gliomas.

In the present study, we comprehensively explore the connections of the expression of lactate metabolism-related genes with the clinical, pathological and immunological aspects of gliomas. However, there were still some limitations in this study. First, in spite of the usage of up to three independent data sets, the sequencing protocols and data preprocessing steps were different, therefore the PLMRS cutoffs and nomograms were established separately in each cohort. Nevertheless, the major trends identified in the study should not be influenced by the lack of consistency in these parameters. Secondly, in this study, the interactions between expression of LMGs and tumor immunity were derived from *in silico* analysis. The precise effects of different LMG expression profiles on the glioma immune TME and the mechanisms mediating these effects should be elaborated in the future experimental studies.

## Conclusion

In conclusion, we found that the expression signature of LMGs could stratify gliomas into two consensus clusters which exhibited differential clinical, pathological, molecular characteristics, as well as the prognosis of the patients. Gliomas in two LMG expression clusters displayed distinct tumor immune microenvironment landscapes that could potentially imply their response to immunotherapies.

## Data availability statement

The datasets presented in this study can be found in online repositories. The names of the repository/repositories and accession number(s) can be found in the article/**Supplementary Material**. The sequencing data generated in this study are available at the Genome Sequence Archive for Humans (accession code HRA002839). The website of the Genome Sequence Archive for Humans is https://ttps://ngdc.cncb.ac.cn/gsa-human.

## Ethics statement

The studies involving human participants were reviewed and approved by Ethical Committee of Sichuan University. The patients/participants provided their written informed consent to participate in this study.

## Author contributions

Study design: ZW, SZ, YL, and MC; data retrieve: JL, SC, YY, WL, and ZW; statistical analysis: SZ and MZ; result interpretation: ZW, SZ, and WF; writing-original draft: all authors; writing-revise: YL, MC, and SZ. All authors contributed to the article and approved the submitted version.

## Funding

This study is supported by Clinical Research Innovation Project, West China Hospital, Sichuan University (19HXCX009 to YL), Post-Doctor Research Project, West China Hospital, Sichuan University (20HXBH035 to SZ), Science and technology project, technology innovation research and development project, Chengdu (2022-YF05-01456-SN to MC).

## Acknowledgments

Our sincere thanks to the developers and maintainers of TCGA, CGGA, the R software contributors, and all the related algorithms and websites.

## Conflict of interest

The authors declare that the research was conducted in the absence of any commercial or financial relationships that could be construed as a potential conflict of interest.

## Publisher’s note

All claims expressed in this article are solely those of the authors and do not necessarily represent those of their affiliated organizations, or those of the publisher, the editors and the reviewers. Any product that may be evaluated in this article, or claim that may be made by its manufacturer, is not guaranteed or endorsed by the publisher.

## Footnotes

1
https://portal.gdc.cancer.gov/

2
http://www.cgga.org.cn/index.jsp

3
https://cibersortx.stanford.edu/

4
http://cbioportal.org

5
http://www.R-project.org
