# Supplementary material for: Prognostic value of lactate metabolism-related gene expression signature in adult primary gliomas and its impact on the tumor immune microenvironment
Source: Front Oncol. 2022 Sep 20;12:1008219. doi: 10.3389/fonc.2022.1008219 (PMC9530666; doi:10.3389/fonc.2022.1008219)
Supplement: Supplementary file 2 [file DataSheet_2.docx]

Supplementary Material

***Patient recruitment and Sample collection of WCH***

Glioma patient between January 2018 and October 2020 in Department of Neurosurgery of West China Hospital, Sichuan University were enrolled in this study. Tumor samples were collected during surgical procedure. Pathological diagnosis was made according to the 2016 World Health Organization (WHO) Classification of Tumors of the Central Nervous System(1). Two experienced neuropathologists participated in the diagnosis and grading of each case. Inclusion criteria are: 1)Age ≥ 18 years ;2)Male or non-pregnant female; 3)Receiving elective craniotomy for intracranial mass lesion; 4)Any type of primary glioma in formal pathologic diagnosis report; 5)No history of any other malignant, neurological, significant infectious or rheumatic disease.

***RNA extraction and sequencing***

Sequencing glioma tissues were derived from surgical specimens, whose size was close to a 4-millimeter cube. Then samples were washed with saline and frozen in liquid nitrogen within 30 minutes. Trizol reagent (Invitrogen, USA) was used for RNA isolation, according to the manufacturer’s instruction. NEBNext® UltraTM RNA Library Prep Kit for Illumina® (NEB, USA) was used to construct sequencing libraries. Quality check of library was carried out on the Agilent Bioanalyzer 2100 system. The clustering of the index-coded samples was performed by TruSeq PE Cluster Kit v3-cBot-HS (Illumia) on a cBot Cluster Generation System, according to the manufacturer’s instructions. Sequencing was performed by Illumina Novaseq S6000 platform and 150 bp paired-end reads were generated. Clean reads were mapped and counted by STAR.

***Reference***

1. Louis DN, Perry A, Wesseling P, Brat DJ, Cree IA, Figarella-Branger D, et al. The 2021 Who Classification of Tumors of the Central Nervous System: A Summary. *Neuro Oncol* (2021) 23(8):1231-51. Epub 2021/06/30. doi: 10.1093/neuonc/noab106.

Supplementary Table 1 Number of genes in each lactate metabolism related pathway of MSigDB.

Supplementary Table 2 Differentially expressed genes between adult primary gliomas and normal brains in the TCGA cohort.

Supplementary Figure 1 CDF of consensus clustering.

Abbreviation: CDF, cumulative distribution function.

Supplementary Figure 2 Nomogram of adult primary gliomas in CGGA and WCH cohort. (A) Nomogram of adult primary glioma in CGGA. (B) Nomogram of adult primary glioma in WCH.

Abbreviation: CGGA, Chinese Glioma Genome Atlas; WCH, West China Hospital; IDH, isocitrate dehydrogenase; WHO, World Health Organization; PLMRS, prognostic lactate metabolism risk score.
