# Supplementary material for: Prognostic value of lactate metabolism-related gene expression signature in adult primary gliomas and its impact on the tumor immune microenvironment
Source: Front Oncol. 2022 Sep 20;12:1008219. doi: 10.3389/fonc.2022.1008219 (PMC9530666; doi:10.3389/fonc.2022.1008219)
Supplement: Supplementary file 3 [file DataSheet_3.pdf]

Supplementary Figures

1 Supplementary Figures

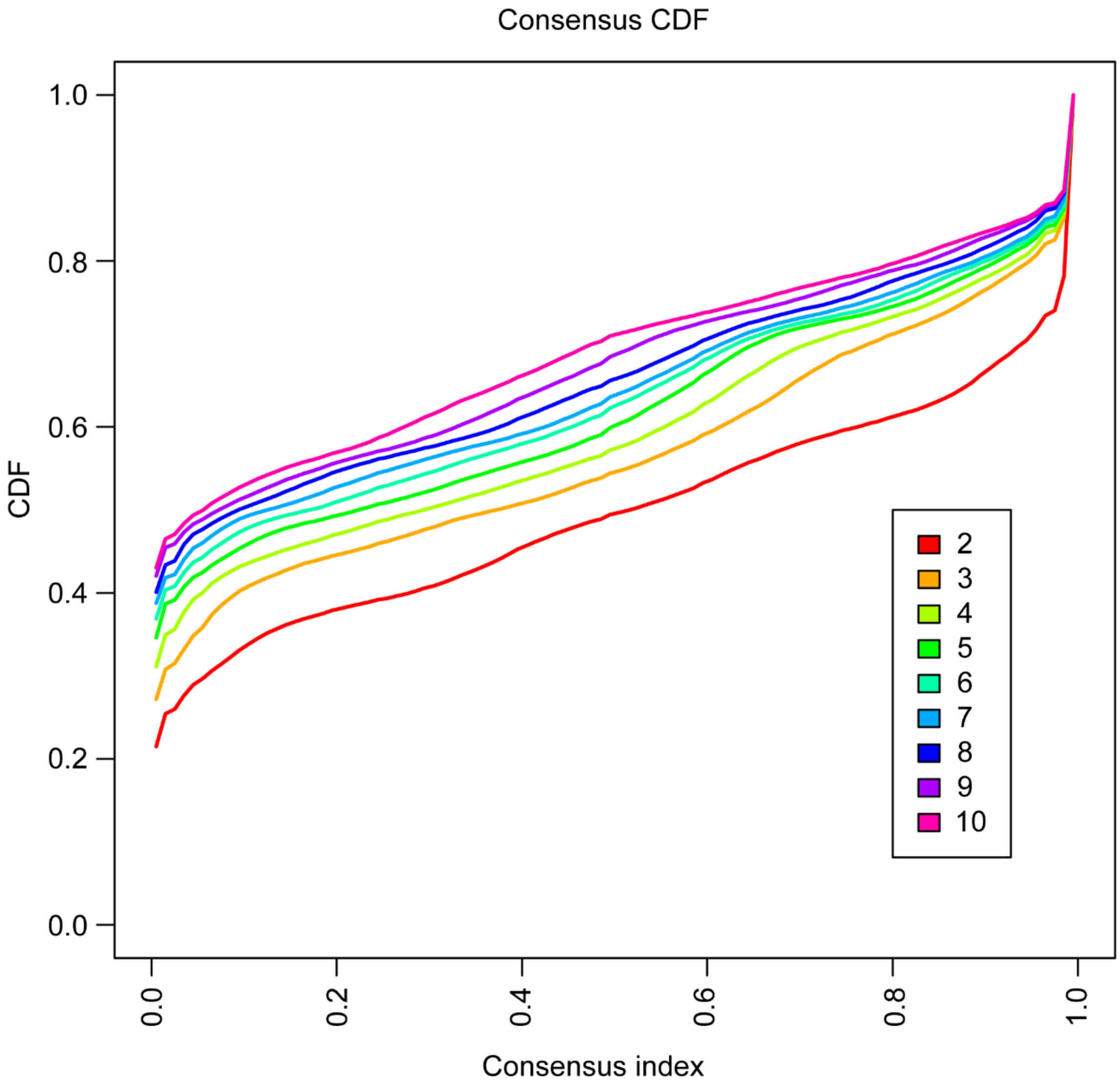

Supplementary Figure 1. CDF of consensus clustering. CDF, cumulative distribution function.

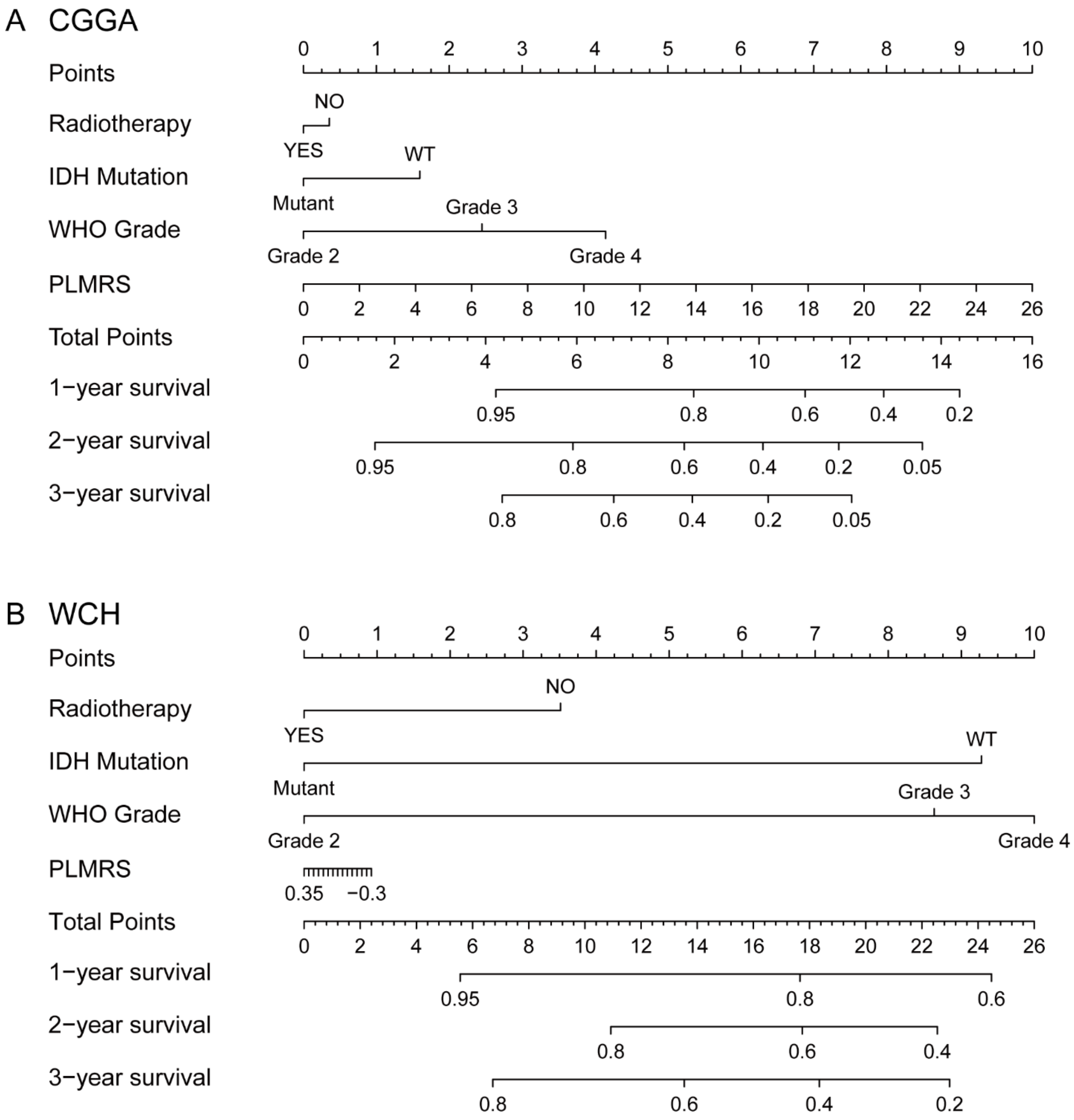

Supplementary Figure 2. Nomogram of adult primary gliomas in CGGA and WCH cohort. (A) Nomogram of adult primary glioma in CGGA. (B) Nomogram of adult primary glioma in WCH. CGGA, Chinese Glioma Genome Atlas; WCH, West China Hospital; IDH, isocitrate dehydrogenase; WHO, World Health Organization; PLMRS, prognostic lactate metabolism risk score.
