# Supplementary material for: Prognostic value of lactate metabolism-related gene expression signature in adult primary gliomas and its impact on the tumor immune microenvironment
Source: Front Oncol. 2022 Sep 20;12:1008219. doi: 10.3389/fonc.2022.1008219 (PMC9530666; doi:10.3389/fonc.2022.1008219)
Supplement: Supplementary file 4 [file Table_1.docx]

Supplementary Table 1 Number of genes in each lactate metabolism related pathway of MSigDB.

| **ID of pathway** | **Name of pathway** | **No.Genes** |
| --- | --- | --- |
|  | Total (Unique Genes) | 205 |
| M35671 | HP_INCREASED_SERUM_LACTATE | 138 |
| M35857 | HP_INCREASED_CSF_LACTATE | 71 |
| M38632 | HP_ABNORMAL_LACTATE_DEHYDROGENASE_LEVEL | 40 |
| M38282 | HP_INCREASED_LACTATE_DEHYDROGENASE_LEVEL | 26 |
| M22371 | GO_LACTATE_METABOLIC_PROCESS | 11 |
| M34151 | GO_LACTATE_TRANSMEMBRANE_TRANSPORT | 5 |
| M38230 | HP_ABNORMAL_BRAIN_LACTATE_LEVEL_BY_MRS | 5 |
| M26344 | GO_LACTATE_TRANSMEMBRANE_TRANSPORTER_ACTIVITY | 4 |
| M26163 | GO_LACTATE_DEHYDROGENASE_ACTIVITY | 3 |
| M34377 | GO_L_LACTATE_DEHYDROGENASE_ACTIVITY | 2 |
